# Hyperelastic Properties of Platinum Cured Silicones and its Applications in Active Compression

**DOI:** 10.3390/polym12010148

**Published:** 2020-01-07

**Authors:** Gayani K. Nandasiri, Anton Ianakiev, Tilak Dias

**Affiliations:** 1 Advanced Textiles Research Group, School of Art and Design, Nottingham Trent University, Bonington Building, Dryden Street, Nottingham NG1 4 GG, UK; 2 Department of Civil Engineering, School of Architecture Design and Built Environment, Nottingham Trent University, Nottingham NG1 4FQ, UK; anton.ianakiev@ntu.ac.uk

**Keywords:** venous disease, compression therapy, active compression, interface pressure, pressure transmission

## Abstract

This paper presents the fundamental research of design, development, and evaluation of an active compression system consisting of silicone based inflatable mini-bladders, which could be used in applying radial pressure for the treatment of venous disease. The use of mini-bladders will nullify the effect of radius of curvature and provide a higher resolution to the pressure distribution. They are designed with two elastomeric layers and inflation is limited only to one side. The mini-bladders apply a radial force onto the treated surface when inflated, and the pressure inside mini-bladders could be measured using the concept of back pressure, which provides the flexibility to inflate mini-bladders to a predefined pressure. The 3-D deformation profile of the mini-bladders was analysed using finite element method (FEM) and FEM simulations were validated with experimental data, which showed good agreement within pressure region required for the treatment of venous disease. Finally, the pressure transmission characteristics of mini-bladders were evaluated on a biofidellic lower leg surrogate and the results have shown that the mini-bladders could apply a uniform pressure irrespective of the location on the leg with a 60%–70% of inlet pressure successfully transmitted onto the leg surface, while 40%–50% was available after the fat layers.

## 1. Introduction

Chronic venous disorder (CVD) comprises of any morphological or functional abnormalities of the venous system, such as varicose veins, oedema, pigmentation, eczema, lipodermatosclerosis, and venous ulcers [1,2,3,4,5]. The most accepted pathophysiologies of venous insufficiency are primary valvular incompetence and vein wall weakness [6,7,8]. CVD, including varicose veins and chronic venous insufficiency is one of the most common chronic medical condition, affecting around 5%–30% of adult population in the developed countries [9,10,11,12,13,14]. The sheer prevalence of varicose veins and substantial costs of treating late complications such as chronic ulcers contribute to a high burden on health systems, which accounts for a 1%–3% of total health care budget of many Western countries [15,16,17].

Compression therapy is considered as the most widely used treatment preventing adverse effects of CVD. The compression therapy aims to decrease the diameter of the veins, which increases the flow velocity, and also improves valvular function providing better coaptation of valve cusps [8,18]. The compression therapy to be effective, a higher pressure should be applied at the ankle which is then graduated towards the knee in order to provide an external pressure gradient that works against the hydrostatic pressure to aid the venous return [3,19,20,21,22,23]. In medical literature the optimum pressure profile for compression therapy that is most commonly described is around 40–45 mmHg at the ankle, reducing gradually to 15–20 mmHg at the knee [24,25]. Current compression therapy takes the forms of medical compression bandages (MCB), medical compression stockings (MCS), and intermittent pneumatic compression (IPC) which can be divided in to two major categories as passive and active compression [26,27,28]. MCBs and MCSs are considered as passive compression treatment systems, which work based on the principle of generating a pressure on the skin surface by a component of the tangential tension developed in an elastic fabric due to its stretch [29]. This generated pressure is proportional to the tension of the fabric and inversely proportional to the radius of curvature of the surface; according to the Laplace’s law [30,31,32]. Intermittent pneumatic compression (IPC) is considered as an active compression system which squeezes the leg, mimicking the action of leg muscle pumps and has a history of being used effectively for treating lymphedema [33,34,35]. Systems used in this application could consist of large single bladder cuffs [36] to complex multi chamber bladder systems employing graded sequential compression [37]. Another field of application for pneumatic compression is the injury treatment for high performance athletes, where bladders are used as pneumatic compression devices. The compression characteristics of such devices are described as peristatic pulse compression (PPC), which was developed by NormaTec [38].

However, human limbs do not have a perfectly circular cross section [29], hence even if MCBs and MCSs would provide a uniform fabric tension, the geometry of the limb will result in pressure variations due to the differences in radius of curvatures. Therefore, the applied pressure will be different at different points of the limb cross section. Also, both theses conventional methods have the disadvantage of losing their elasticity over the time, making the stockings and the bandages to snag resulting in wrong pressure profiles. In order to overcome these limitations in the existing treatment methods, the current study proposes an active compression system which could apply a radial force based on the pressure exerted by a volume of air trapped inside a mini-bladder. The advantage of using mini-bladders is that they can provide an accurately controlled pressure resolution by using multiple mini-bladders in a network of discrete points instead of using large single bladder cuffs. As the pressure inside these mini-bladders could be measured using the principle of back pressure, the system provides a more controllable pressure profile than the existing treatment systems and will pave the way towards developing smart compression systems.

This paper presents, the geometrical analysis of the mini-bladders made from soft silicone elastomers and their 3D deformation profile using FEM. The mini-bladders have also been studied for their effectiveness in terms of the interface pressure transmission on a biofidelic lower leg surrogate. Finally, the mini-bladders have been integrated to produce a final design prototype of an active compression sleeve that could be effectively utilized for the treatment of venous disease and lymphoedema.

## 2. Materials and Methods

There are a number of design characteristics that need to be addressed in order to develop a wearable active compression device that can successfully provide a graduated compression profile. Firstly, the material that is used to cast the mini-bladders should be bio-compatible and should provide good elastomeric properties. The mini-bladders should be designed to achieve maximum interface pressure of 40–50 mmHg on the treated surface. The mini-bladders should then be integrated in a wearable device in a manner that enables a patient to easily put it on and take it off. Also, the overall profile should be kept as thin as possible and lastly, to be effective the mini-bladders should be capable of producing repeatable results.

### 2.1. Material Properties and Characterisation

Polymers are often used in manufacturing bladders for different applications, polyurethane and rubber are the most commonly used in this regard. However, they are considered not bio-compatible as both cause allergic reactions and risk of asthmatic symptoms with direct contact with the skin [39,40,41]. Silicones are considered to be bio-compatible with good elongation properties (around 600%–1100%) and a very good memory [42]. Silicones exhibit non-linear elastic behavior and are considered as hyperelastic materials. In this study platinum cured RTV (Room Temperature Vulcanized) silicones of shore OO 30 (MouldLife, Suffolk, UK, PlatSil^®^ Gel-OO) with an elongation at break (strain) value of 800%–900% was used. The uniaxial tensile tests were carried out according to BS 37: 2011 (Type 1a specimen) with an elastic strain rate of 500 mm/min using a Zwicki 2.5 KN (Zwick Roell, Ulm, Germany) tensile testing machine. Due to the hyperelastic nature of the selected silicone, a Yeoh second order hyperelastic mathematical model was used to describe the material behaviour. 

### 2.2. Mini-Bladder Design and Geometrical Analysis

**Figure 1:**
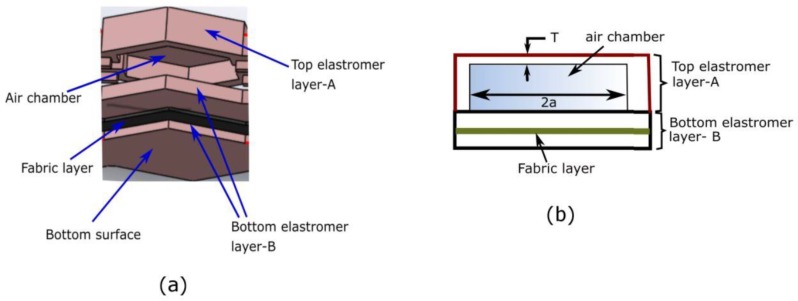
(**a**) The layered view of mini-bladder design, (**b**) schematic of the mini-bladder of consisting layers and the air chambers.

The mini-bladders were specifically designed to have one side of the mini-bladder inflated fully (extensible top layer A), while inflation of the other side was restrained (less extensible bottom layer). For the inextensible layer a woven fabric (151.69 GSM, 62 epi, 60 ppi) of 0.38 mm thickness having a Young’s modulus of 0.964 MPa (calculated according to [43] was embedded into the silicone layer. This allowed to use the energy of the supplied air efficiently, to maximize the pressure transmission onto the contact surface. As shown in the Figure 1a, a less extensible bottom layer (elastomer layer B) was used as the base of the mini-bladder yet was sufficiently flexible to minimize the rigidity of the entire mini-bladder. When pressurized, the top layer of the mini-bladder inflated fully while the bottom layer had less/no inflation. The two layers were sealed from the sides with no inflation in the sides of the mini-bladders.

To determine the relationship between inflation height and the geometry of the mini-bladders, the Yeoh second order curve fitting model was adopted with the experimentally calculated coefficients $C_{1}$ and $C_{2}$. Assuming the incompressibility of the mini-bladder across the thickness, the Yeoh model can be written as;

(1) $$U\;=\;C_{1}\left[\lambda +\frac{1}{\lambda^{2}}-2\right]+C_{2}{\left[\lambda +\frac{1}{\lambda^{2}}-2\right]}^{2}$$

**Figure 2:**
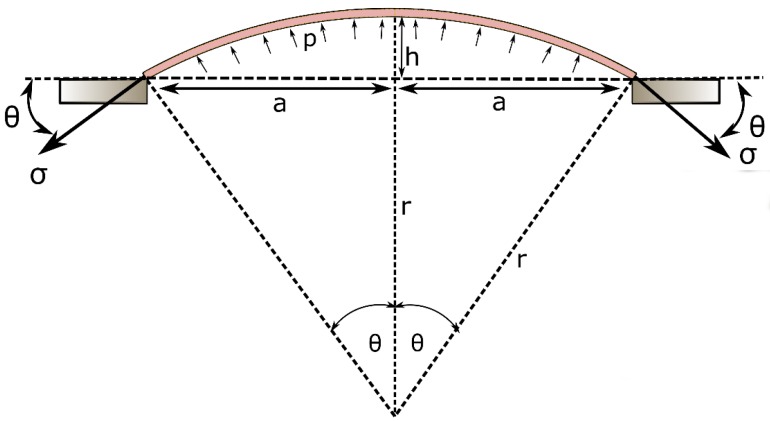
Schematic diagram of the mathematical representation of the inflation of the mini-bladder.

The inflation profile of the top membrane can be theoretically approximated as a hyperplastic membrane with two ends clamped giving an inflation profile as shown in Figure 2. Hence, λ can be written as:

(2) $$\lambda \;=\;\frac{\theta }{\sin\theta }$$

where θ can be calculated as,

(3) $$\theta \;=\;\mathrm{asin}\left(\frac{2ah}{\left(h^{2}+\;a^{2}\right)}\right)$$

Therefore, the stress of the membrane $\sigma \;=\;\frac{\partial U}{\partial \lambda }$ becomes a function of *a* and *h*;

(4) $$\sigma \;=\;2\left[\lambda -\frac{1}{\lambda^{3}}\right]\left[C_{1}+2C_{2}\left(\lambda^{2}+\frac{1}{\lambda^{2}}-2\right)\right]$$

Assuming the inflation profile is a spherical dome, using the force balance, the relationship between σ and the supplied air pressure ΔP can be written as;

(5) $$\sigma \;=\;\frac{\left(h^{2}+a^{2}\right)\cdot \Delta P}{4Th}$$

By solving Equations (2)–(5) using numerical techniques, a relationship between the inflation height (h), membrane thickness (T), and the minimum gap distance between the membrane fixing points (a) could be achieved. This semi-analytical formulation would provide a guideline to design the geometry and the size of the mini-bladders. The size and the thickness of the mini-bladder should be designed such that it should be able to achieve a certain minimum height that is required to establish a sufficient contact between skin and the compression sleeve. The smaller membrane thicknesses would provide higher inflation heights (Supplementary Figure S1), however to avoid the rupture and increase the stability the top membrane thickness was selected as 1.5 mm. The inflation height shows a positive correlation with minimum gap between the membrane fixing points (Supplementary Figure S1). According to the geometry, circular shaped mini-bladders had the largest minimum gap distance while hexagonal shaped mini-bladders has a slightly lower value (Supplementary Table S1). Hence, to obtain a higher inflation height, compared to square shaped mini-bladders, either hexagonal or circular shaped mini-bladders could be used. However, the hexagonal shaped mini-bladders would give the most efficient packing by arranging them into a honeycomb structure. Hence, the hexagonal shaped mini-bladders of 8 mm side length was selected for initial testing, as downsizing more than 8 mm would make errors in manufacturing due to the manual assembly of the mini-bladder unit, also upscaling the size of the mini-bladders would lose the advantage of using mini-bladders over large sized bladders. 

### 2.3. Pressure Transmission of Mini-Bladders on a Biofidelic Leg Surrogate

**Figure 3:**
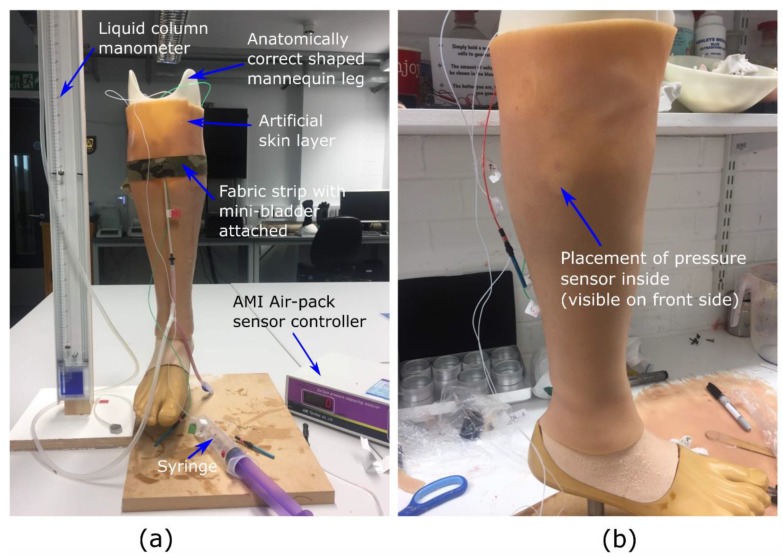
The experimental set up used to evaluate the pressure transmission efficiency, (**a**) measurement set up (**b**) the AMI. senor embedded in the fat layer visible through the skin layer.

The research hypothesis suggests that the proposed active compression system using inflatable mini-bladders should be able to generate uniform pressure, irrespective of the position of the leg. Therefore, to test this hypothesis it was decided to measure the interface pressure generated by the mini-bladders at four different positions of the biofidelic lower leg surrogate, namely anterior (front), posterior (back), lateral (outward side) and medial (inward side). If the interface pressures recorded at these four positions are the same, it could be concluded that the research hypothesis is proven. It was also decided to study the effect of the pressure transmission through the layers of the skin as this would provide a better understanding on the propagation of the skin contact pressure in the leg; which has not been studied in the context of compression therapy. The pressure through the skin was measured by embedding AMI Air-pack sensors of 20 mm diameter (Model 3037; AMI Techno, Tokyo, Japan) in the fat layer according to the distances corresponding to anterior, posterior, lateral and medial positions at the knee region. Another set of AMI air-pack pressure sensors were placed on the surface of the skin layer aligned with the AMI air-pack sensors embedded in the fat layer (Figure 3b) to measure the interface pressure applied by an inflated mini-bladder. 

The skin is found to be anisotropic and viscoelastic with a range of Young’s modulus between 5 kPa and 140 MPa [44], which is a quite a higher range. Hence, it was decided to manufacture a skin and a fat layer which was having the modulus values around 0.1–1 MPa, using PDMS silicones which closely resembles the tactile properties of the skin. The sensors were calibrated according to the standard procedure recommended by the manufacturer and validated against the TJ600 vertical column liquid manometer (measuring range of 0–130 mbar, and Volt 1S manometer liquid of 1.86 density, KIMO Instruments, Kent, UK) and standard weights.

The mini-bladder was stitched into a woven fabric having a Young’s modulus of 1.648 MPa. Here, a fabric with higher young’s modulus was selected to have a comparatively less extension than the silicon layer, to minimize effect of additional pressure created due to the stretch of the fabric. The mini-bladders were inflated in 2.0 mL of air volume increments with 30 s hold at each increment to record the interface pressure data, while the pressure inside the mini-bladders were measured using the manometer. The tests were repeated for five cycles for five samples at each position and the average values for the samples were included in the results. 

### 2.4. Characterisation of 3D Deformation Profile of Mini-Bladders

**Figure 4:**
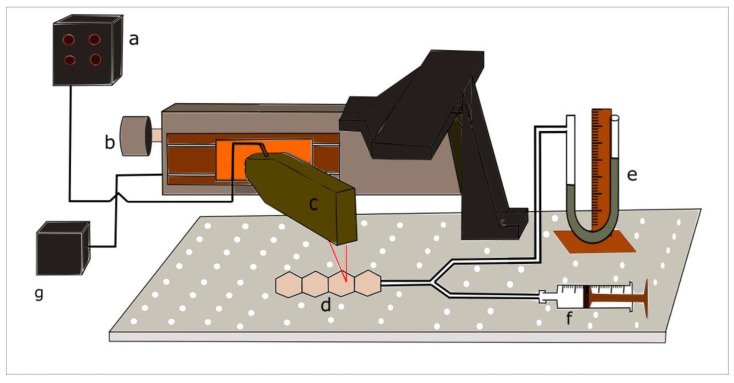
Experimental setup for measuring the inflation height against input air pressure (**a**) Height measurement display panel and controller (**b**) linear translation stage (**c**) laser measurement head (**d**) inflatable hexagonal mini- bladder unit (**e**) liquid manometer (**f**) syringe (**g**) power supply.

After designing and manufacturing the individual mini-bladders of hexagonal shape, for the experimental analysis a unit consists of a four internally connected mini-bladders was manufactured. The experimental study was conducted using such mini-bladder unit to resemble the actual operation where a cluster of mini-bladders will be used in a network. The experimental platform developed to characterise and quantify the 3D deformation profile of mini-bladders with respect to their inflation deflation height is shown in Figure 4. The test rig consists of a laser measurement head LK-G 82 (Keyence, Milton Keynes, UK) having a red semiconductor, class II laser of 650 mn wavelength with a measuring range of 30 mm, with an accuracy of 0.1 μm. It was fixed to a 150 mm linear translation stage, with integrated stepper motor controller (Thorlabs, Ely, UK), having 5 μm on-axis accuracy. The laser measurement head was connected to LK-G3000 all in one controller (Keyence, UK) with the LK-Navigator support software which was used to display and store the measured data. The mini-bladder unit was tied onto the bread board using thin cords to secure its position and was connected via a Y connector to a vertical liquid column manometer. The manometer was used to measure the inlet air pressure. 

For the height measurement LK-G 82 laser measurement head was fixed onto the linear stage so that it could move along the centre axis of the mini-bladder unit. The LK-G 82 was fixed, such that the laser point coincides with the centre line position of each mini-bladder. Then LTS 150 stage could be accurately moved from the mid-point of one mini-bladder to the next by specifying the distance on the software. The air volume inside the bladders were increased by 2 mL increments and after inflating the mini-bladders to a certain pressure, it was kept for 1 min to stabilise before the mid-point height measurement was taken for each component mini-bladder. Each test was repeated for five cycles to evaluate the accuracy and the repeatability of the results.

### 2.5. FEA Analysis of 3D Deformation Profile of Mini-Bladders

A FEM approach was selected to study the 3D deformation of the mini-bladders, this allows to predict the deformation of each mini-bladder in the unit and compare the results with the experimental data obtained. The FEA analysis provide a detailed view of the 3D deformation profile of the individual mini-bladders providing any disruptions that could occur to the adjacent mini-bladders, during the inflation of each mini-bladder. The mini-bladders were treated with standard/implicit FEM using ANSYS Workbench (ANSYS Academic, 18.1 version, ANSYS, Inc., Canonsburg, PA, USA). A mesh of 1 mm resolution was used for the analysis, as 1 mm resolution was deemed to be an appropriately high resolution for the mini-bladder unit that was intended to be ~50 mm across length. The Yeoh second order model was used to model the 3D deformation of the mini-bladders with the material constants obtained under Section 2.1. The boundary conditions were applied to reflect the experimental conditions described in Section 2.3. During the FEM validation, the membrane thickness and minimum gap distance were also examined under several simulations and their results are included in Supplementary Figures S2 and S3.

## 3. Results and Discussion

### 3.1. Model Parameters for Hyperelastic Equations

As described above, due to the hyperelastic nature of the selected silicone, a Yeoh model was used to characterize the material constants as it is capable of predicting the stress strain behaviour for different deformation modes even with the data obtained from only one simple mode of deformation like uniaxial extension [45]. The uniaxial tensile test data was fitted to the Yeoh second order model (Equation (1)) using MatLab curve fitting tool (version: MatLab R2016b, MathWorks^®^ Inc., Natick, MA, USA) (Figure S4, see Supplementary Material). It can be seen from the experimental data that the Yeoh second order model provides a good fit with model coefficients of $C_{1}$ = 0.04957 MPa, and $C_{2}$ = 0.0000565 MPa (coefficient of determination R^2^ = 0.995, and root mean square error RMSE = 0.022).

### 3.2. 3-D Deformation Profile Results of the Mini-Bladders

**Figure 5:**
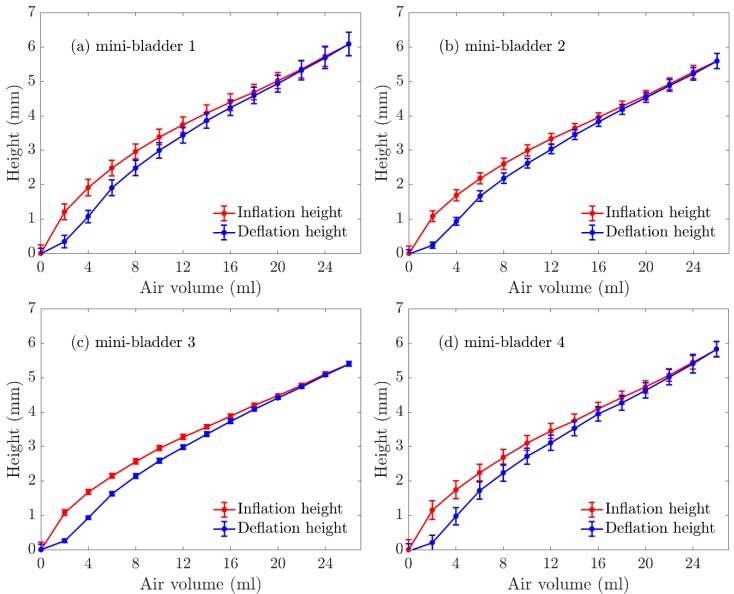
Experimentally measured inflation deflation height (average) of mini-bladder unit of 4 hexagonal mini-bladders made of PlatSil^®^ Gel-OO. (**a**) mini-bladder 1, (**b**) mini-bladder 2, (**c**) mini-bladder 3, (**d**) mini-bladder 4.

The 3-D deformation profile of the mini-bladders were experimentally observed as discussed in Section 2.4 and the maximum inflations heights were measured using leaser measurement head. The inflation height was measured in both inflation and deflation cycles and the results for the mini-bladder inflation heights for four individual mini-bladders in a mini-bladder unit are shown in Figure 5.

The average inflation height varied between 0–7 mm; while mini-bladder 1 had the highest inflation, followed by mini-bladder 2 and 3. However, mini-bladder 4 showed slightly higher inflation height than mini-bladders 2 and 3. This may have been caused due to the method used to secure the mini-bladder unit during the experiment. It can also be noted the that the deviations between the tested samples were low.

**Figure 6:**
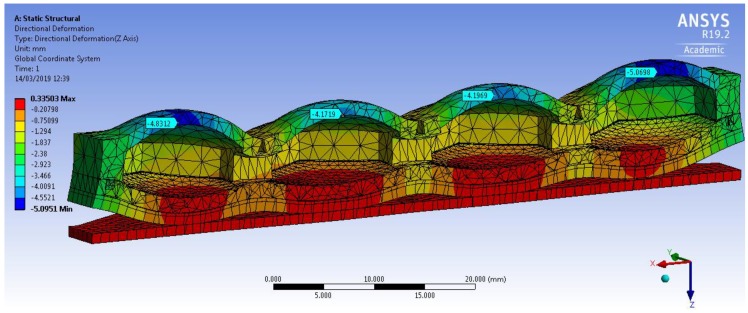
Simulation of the directional deformation (deformation in z direction) of PlatSil^®^ Gel-OO hexagonal mini-bladder unit for pressure of 81 mmHg. The maximum deformation is shown in the legend.

The Yeoh second order model and the empirically derived above parameters were used for FEM simulations. The 3D deformation profile of the mini-bladder unit under an internal pressure of 81 mmHg is shown in Figure 6. The deformation range is indicated by the color map. The simulation results clearly indicate that slight curling occurred at the ends of the mini-bladder unit. Similar behavior is also observed in the experiments. This happened due to the fact that mini-bladder unit is not fixed to the bread board at the end due to the practical difficulties during the experiments.

**Figure 7:**
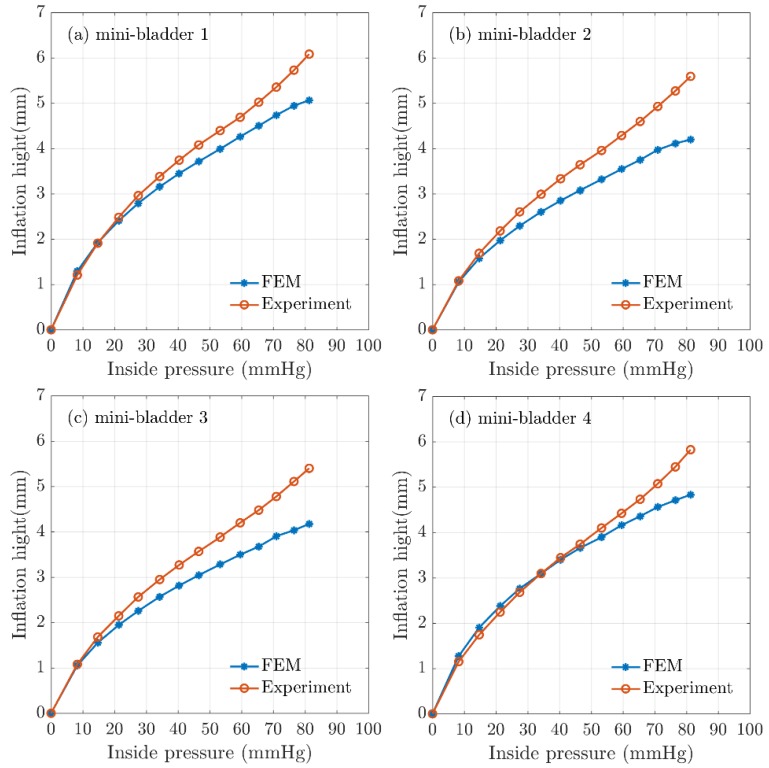
Comparison of FEA results for inflation height and experimental mean height for hexagonal mini-bladder unit against the pressure between 0mmHg and 85 mmHg. Experimental data are shown in orange (mean height values) and the model values shown in blue for (**a**) mini-bladder 1, (**b**) mini-bladder 2, (**c**) mini-bladder 3, and (**d**) mini-bladder 4.

The mean experimental results of the maximum inflation-deflation height for hexagonal mini-bladder units of 8 mm side length made of PlatSil^®^ Gel-OO 30 was compared against the FEM results obtained for the highest directional deformation at each pressure value used for the inflation (Figure 7). It can be seen from Figure 7 that the FEM results for all the mini-bladders showed more than 80% agreement with the experimental data for the range of inside pressures from 0–60 mmHg. This pressure region corresponds to the optimum interface pressure for the compression therapy in the literature. The results demonstrated that the model gave a good representation of the physical system in this pressure range. As can be seen from Figure 7, model results started to deviate from experimental values at pressures above 70 mmHg.

The boundary conditions of the simulations were set to reflect the experimental conditions; however, there can be approximation errors of parameters like the friction coefficient which was deduced from the data available in the literature. The frictional forces could have a significant impact in high pressure conditions due to the occurrence of slippage. It is possible to achieve higher inflation heights by reducing the membrane thickness (Supplementary Figure S3), however reducing the membrane thickness will have effects on membrane rupture and instability. Hence, a membrane thickness of 1.5 mm was decided to use as the final prototype thickness since the mini-bladders made with this thickness provided efficient results. The FEM simulations carried out for different hexagonal geometrical sizes and membrane thicknesses shown that also increasing the size of the mini-bladders and reducing the membrane thickness have positive impact on the inflation height (Supplementary Figures S3 and S4).

### 3.3. Interface Pressure Transmission Results

**Figure 8:**
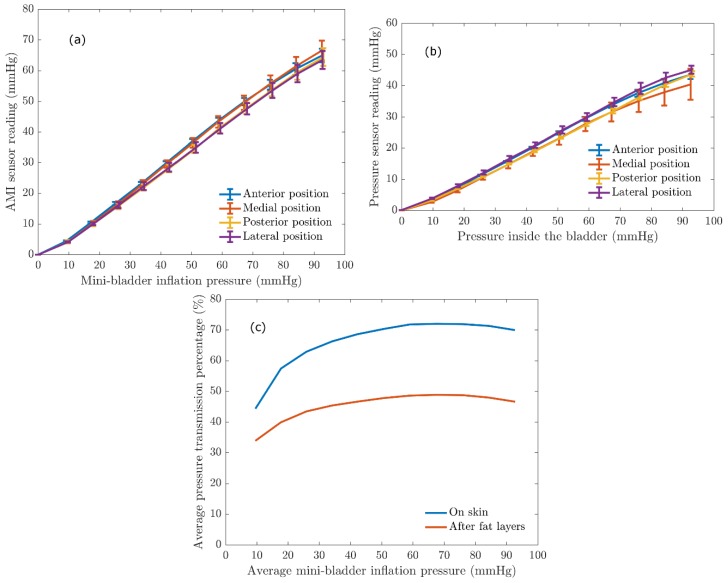
The relationship between the AMI sensor reading and the mini-bladder inflation pressure. (**a**) interface pressure on the artificial skin layer (Young’s modulus 0.22 MPa) (**b**) the pressure recorded by AMI air-pack sensor embedded in the fat layer (Young’s modulus 0.03 MPa) (**c**) Average pressure transmission percentage against the average mini-bladder inflation pressure, for outer skin layer and the inside fat layer of the leg surrogate.

It is important to quantify the pressure transferred via the mini-bladders onto a biofidelic lower leg surrogate to evaluate the efficiency of the mini-bladder performance, as it will depict the inflatable mini-bladders in contact with the human skin to deliver the required pressure profile and also to study the propagation of the pressure through the layers of the skin. This study of propagation of the pressure through the layers of skin was a gap in knowledge in the current compression therapy research. In the present study an experimental platform discussed under 2.3 was used to quantify the pressure transmission properties of the mini-bladders by expressing the interface pressure as a function of the pressure inside the mini-bladders measured as the back pressure using a liquid column manometer. The results obtained for the interface pressure on surface of the artificial skin layer, after fat layers, and pressure transmission efficiency against the mini-bladder inside pressure are shown in Figure 8. The different coloured lines show the interface pressure at anterior, posterior, lateral, and medial positions of the leg surrogate in Figure 8a,b.

It is evident from the results shown in Figure 8 that irrespective of the position of the leg surrogate the interface pressure values were the same with a maximum variation of 2.5 mmHg. This maximum variation is not that significant considering the range of mini-bladders inflation pressures. Therefore, it can be concluded that the mini-bladders can be used to apply a uniform circumferential interface pressure, which is not possible with current passive compression products such as stretch bandaging, compression sleeves and stockings. It was also evident from Figure 8a that the interface pressure and the mini-bladder inflation pressure continued to demonstrate a linear relationship (for all positions measured). This linear relationship can be used as a calibration curve, to find out the pressure to which the mini-bladder should be inflated in order to obtain the required interface pressure. 

A similar relationship was observed with the AMI air-pack sensors embedded in the fat layer. As shown in Figure 8b, the pressure lines for anterior, posterior, lateral and medial positions closely follow each other with a maximum variation of 3 mmHg in the interested pressure range for compression therapy. Therefore, it again confirms that the pressures generated by the mini-bladders were independent of the position of the leg surrogate. Therefore, it can be concluded that the proposed active compression system using mini-bladders can deliver a uniform circumferential pressure irrespective of the radius of curvature. In order to quantify the transmission of the pressure applied by the mini-bladder through skin and fat layers the pressure transmission percentages on the skin and fat layer were calculated using Equation (6), and the obtained results are shown in Figure 8c.

(6) $$Pressure\;transmission\;percentage\;=\;\frac{Interface\;pressure\;\left(mmHg\right)}{mini-bladder\;inside\;pressure\;\left(mmHg\right)}\;\times 100\%$$

As shown in Figure 8c, 50%–70% of the pressure applied by the mini-bladder would be transmitted onto the surface of the leg surrogate (i.e., artificial skin surface). It also demonstrates that the pressure transmission percentage in the fat layer is in the range of 35–45% of the mini-bladder pressure; i.e., in a clinical context only about 35%–45% of the force applied onto the surface of the skin (in this case by mini-bladders) of a human leg would be available after the fat layer to compress the veins. This finding is important for compression treatment systems in estimating the pressures to be applied at the skin surface of a human limb in order to achieve an effective compression of the veins for the treatment of venous return. The results show that, even though a higher skin interface pressure is recorded, the pressure inside the leg would be much lower. Hence, one could propose the use of higher skin interface pressures during the treatment of venous return. However, this would depend on the stiffness of the skin, fat muscle layer modulus and must be considered in deciding the inflation of the mini-bladders in a clinical practice, which will be a future scope of this research.

## 4. Conclusions

This study reports fundamental research towards the design, development, and evaluation of an active compression system consisting of inflatable mini-bladders for the treatment of venous disease. The mini-bladder design was geometrically analysed and validated with a FE model. Uniaxial tests were carried out to understand the mechanical properties of the materials used to manufacture the mini-bladders and those parameters were used in FEM simulations to analyse the 3D deformation behaviour of the inflatable mini-bladder units. The FEM simulation results agreed well with the observed data, where numerical model results for inflation/deflation heights showed more than 80% agreement with experimental data for the inlet pressure range of 0–60 mmHg which is the interested pressure region for the compression therapy.

The pressure transmission efficiency evaluation of the biofidelic lower leg surrogate made with artificial skin and fat layers having Young’s modulus values closer to the actual human skin, fat obtained from the literature (0.22 MPa and 0.03 MPa) has shown the pressure propagation through the artificial skin layer of the leg surrogate was about 50%–70% of the mini-bladder inflation pressure. However, the pressure propagated through the fat layer was around 35%–45% of the mini-bladder inflation pressure. The results have also concluded that mini-bladders were able to apply a uniform circumferential interface pressure irrespective of the position of the leg, which proves the research hypothesis. This is very promising as it is not possible to achieve uniform circumferential pressure with the current passive compression products in the market.

Future steps involve the studying of the pressure transmission behaviour on the leg profile with different types of skin and fat layers. Finally, an evaluation of the performance of the final prototype design (Figure S5), and its extension onto a smart wearable active compression device with the pneumatic controller unit, are anticipated, along with extended evaluation to clinical trials.

## Supplementary Materials

The following are available online at https://www.mdpi.com/2073-4360/12/1/148/s1, Figure S1: Variation of inflation height with (**a**) the membrane thickness, (**b**) minimum gap distance, Figure S2: FE simulations of membrane height variation with different membrane thicknesses of hexagonal mini-bladders, Figure S3: FE simulations for membrane height variation with different sizes of the hexagonal mini-bladders, Figure S4: Experimental and theoretical stress-strain curves, and Figure S5: Final prototype design. Table S1: The relationships between the geometry of the mini-bladders and the minimum gap distance.
