# Supplementary material for: Hyperelastic Properties of Platinum Cured Silicones and its Applications in Active Compression
Source: Polymers (Basel). 2020 Jan 7;12(1):148. doi: 10.3390/polym12010148 (PMC7022899; doi:10.3390/polym12010148)
Supplement: Supplementary file 1 [file polymers-12-00148-s001.pdf]

polymers

Supplementary material

Hyperelastic Properties of Platinum Cured Silicones and its Applications in Active Compression

Gayani K. Nandasiri [1,*], Anton Ianakiev[2] and Tilak Dias[1,*]

1 Advanced Textiles Research Group, School of Art and Design, Nottingham Trent University, Bonington Building, Dryden Street, Nottingham, NG1 4 GG, UK. ; hewa.nandasiri2015@my.ntu.ac.uk (G.K.N); tilak.dias@ntu.ac.uk (T.D)

2 Department of Civil Engineering, School of Architecture and Built Environment, Nottingham Trent University, Nottingham, NG1 4FQ, UK; anton.ianakiev@ntu.ac.uk (A.I)

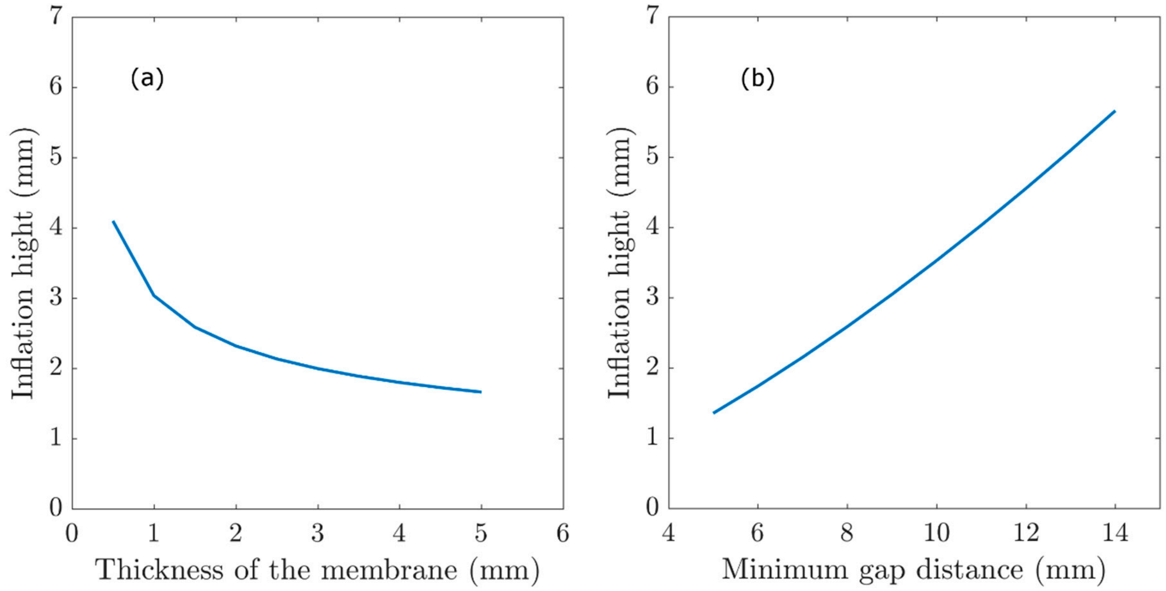

Figure S1: The relationship between inflation height and (a) the thickness of the membrane for hexagonal mini-bladder of 8mm side length (b) minimum gap distance at 5400Pa pressure inside the mini-bladder

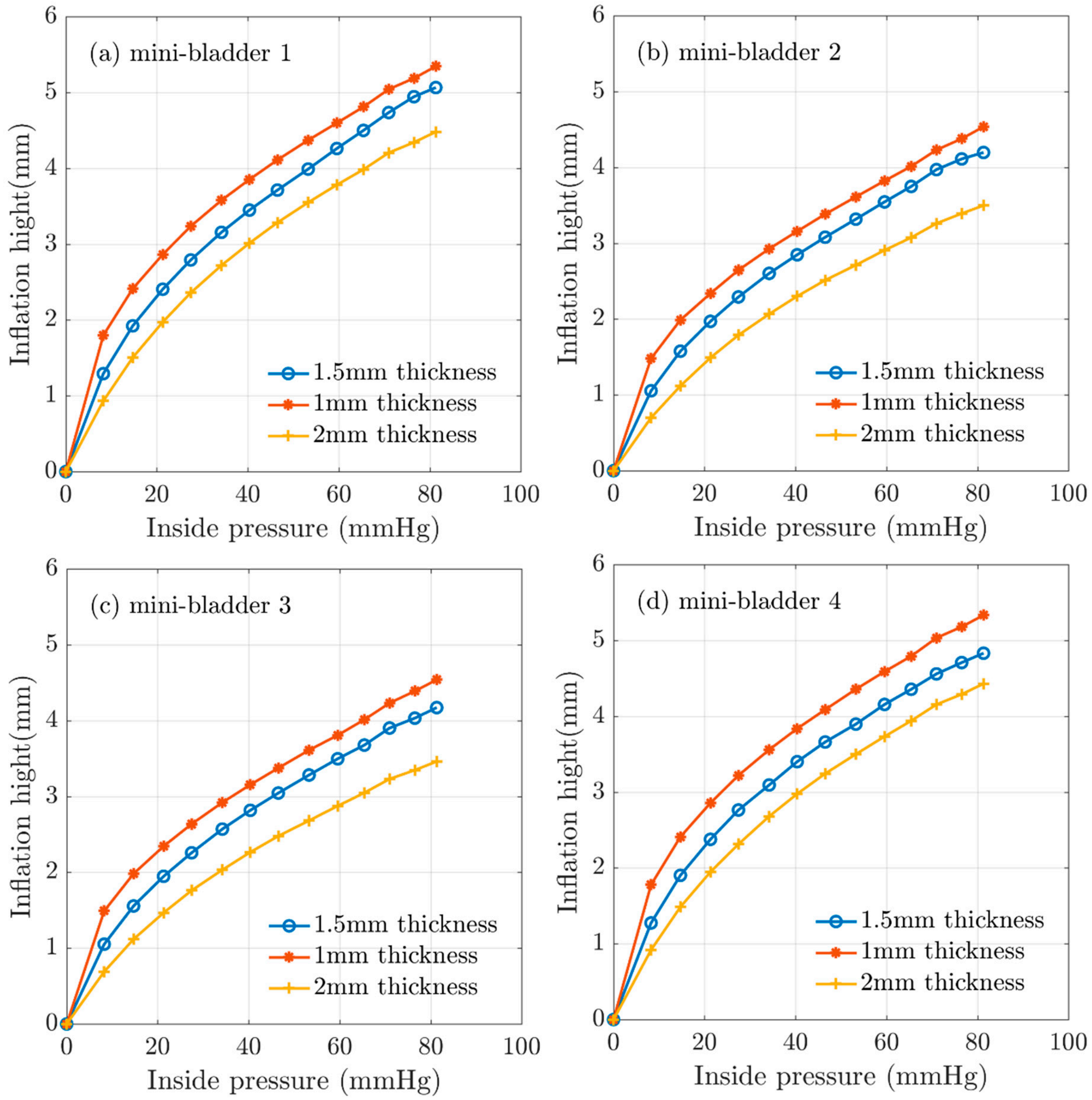

Figure S2: FE simulations obtained for inflation height for different membrane thicknesses for hexagonal mini-bladders of 8mm side length. (For 0-85mmHg pressure inside the mini-bladders).

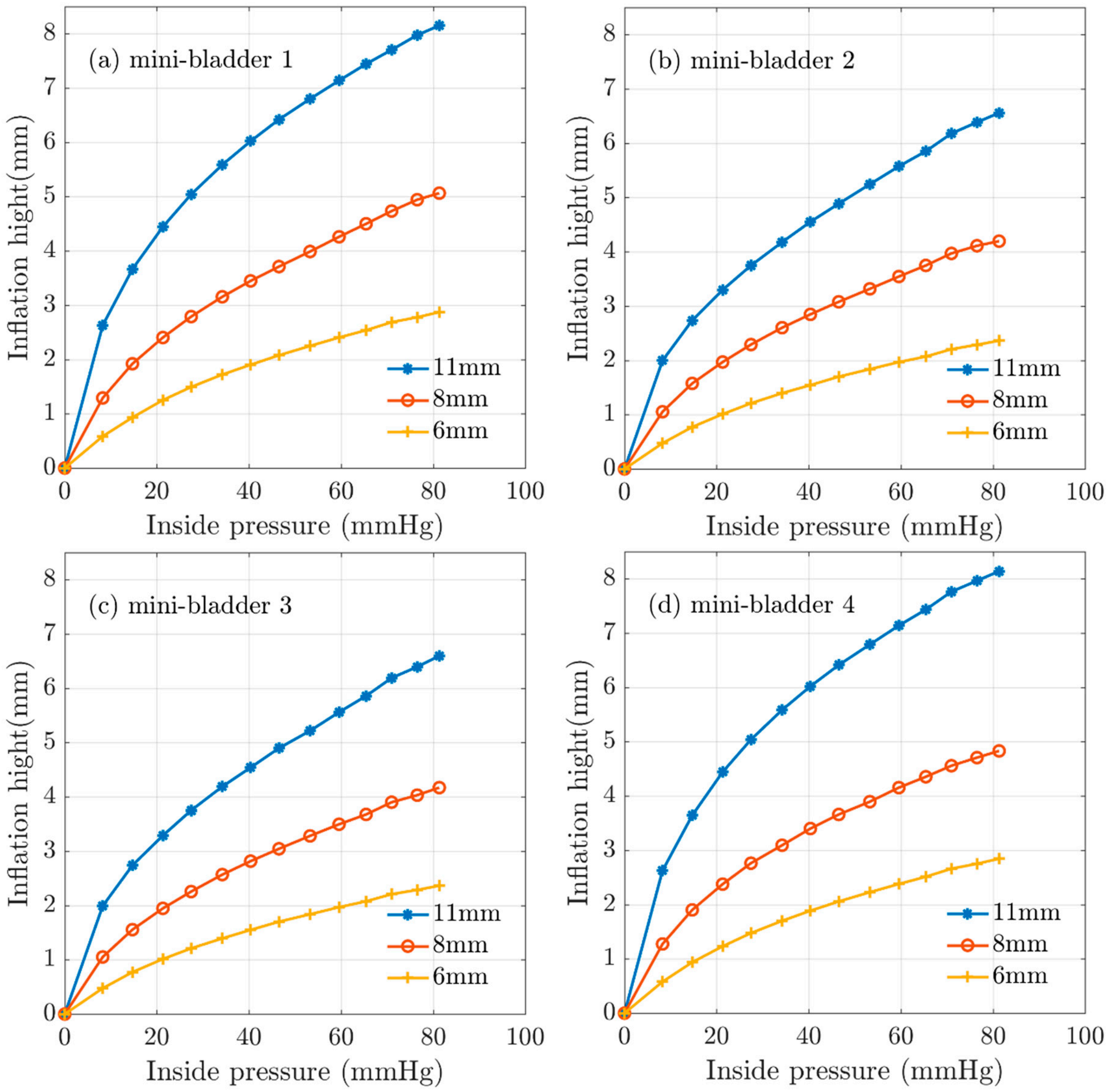

Figure S3: FE simulations obtained for inflation height for different sizes of hexagonal shaped mini-bladders having 1.5mm membrane thickness. (For 0-85mmHg pressure inside the mini-bladders).

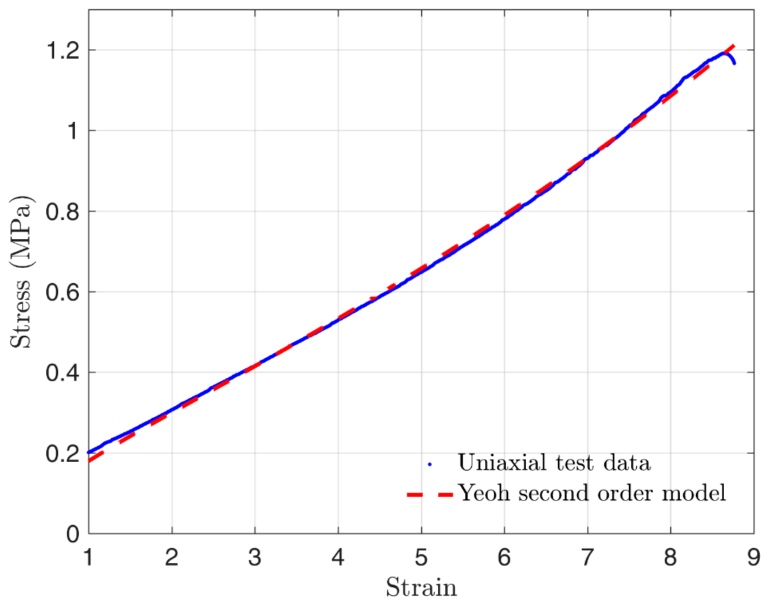

Figure S4: Experimental and theoretical stress-strain curves for the hyperelastic PlatSil® Gel-OO silicone with shore OO 30 hardness. Red dashed line shows the Yeoh second order model with C_1=0.04957 MPa and C_2=0.0000565MPa.

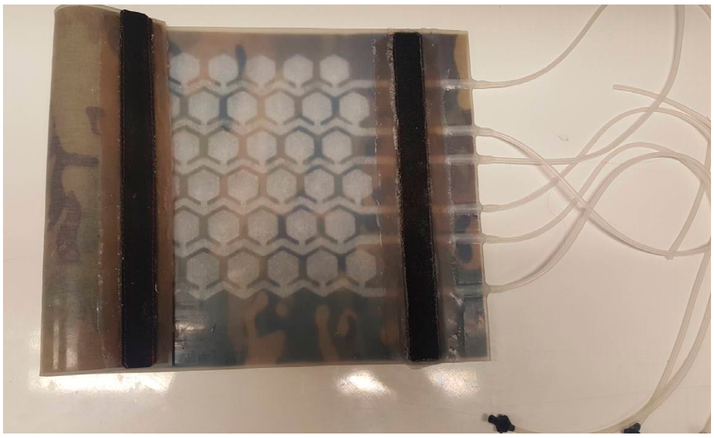

Figure 5: The final prototype of the manufactured active compression sleeve

Table 1: The relationships between the geometry of the mini-bladders and the minimum gap distance (2a)

Type of mini-bladder	Minimum gap distance (mm)
Hexagonal	$\sqrt{3}a$ $=11\sqrt{3}\ = 19.052$
Circular	D= 20.06
Square	a= 17.7 mm

© 2019 by the authors. Licensee MDPI, Basel, Switzerland. This article is an open access article distributed under the terms and conditions of the Creative Commons Attribution (CC BY) license (http://creativecommons.org/licenses/by/4.0/).
